# Amblyopia and Routine Eye Exam in Children: Parent’s Perspective

**DOI:** 10.3390/children8100935

**Published:** 2021-10-18

**Authors:** Alhanouf Alatawi, Naif Alali, Abrar Alamrani, Faris Hashem, Seham Alhemaidi, Shaker Alreshidi, Hani Albalawi

**Affiliations:** 1 Department of Ophthalmology, King Fahad Specialist Hospital, Tabuk 47717, Saudi Arabia; alhanouf.abdullah96@gmail.com; 2 Ophthalmology Division, Department of Surgery, Faculty of Medicine, University of Tabuk, Tabuk 71491, Saudi Arabia; nmalali@ut.edu.sa (N.A.); fhashem@ut.edu.sa (F.H.); salhemaidi@ut.edu.sa (S.A.); 3 Department of Ophthalmology, King Khaled Hospital, Tabuk 47915, Saudi Arabia; abrarmarwan97@gmail.com; 4 Department of Ophthalmology, Faculty of Medicine, Majmaah University, Majmaah 11952, Saudi Arabia; dr.shaker.alreshidi@gmail.com

**Keywords:** amblyopia, parent’s perspectives, knowledge, attitudes, children’s routine eye exams, decrease vision, Saudi Arabia

## Abstract

Amblyopia is a reduced best-corrected visual acuity of one or both eyes that cannot be attributed to a structural abnormality; it is a functional reduction in the vision of an eye caused by disuse during a critical period of visual development. It is considered the leading cause of visual defects in children. With early diagnosis and treatment, children with amblyopia can significantly improve their vision. However, if it is neglected and not treated during childhood, unfortunately, it permanently decreases vision. Therefore, prevention, detection, and treatment largely depend on parents. This article explores parents’ perspectives on amblyopia and routine examination of their children’s eyes. A cross-sectional study used an electronic questionnaire consisting of five main sections to assess the level of awareness of amblyopia among parents. As a result, a total of 325 participants were included in our analysis. 209 (64.3%) were mothers, and 116 (35/7%) were fathers. The age groups were 35–50 years of age (61.5%), 20–34 years (23.4%), and older than 50 years (15%). Participants with a history of amblyopia numbered 23 (7.1%), and 39 had an amblyopic child (12%). A good awareness level of amblyopia among parents was found in only 10 (3%) participants, a fair awareness level in 202 (62%), and 113 (35%) participants were classified as having a poor awareness level of amblyopia. Only 13.8% of the parents took their children for yearly routine eye exams, while the majority (72%) took their children only if they had a complaint, and 14.2% took them for eye checkups only before school entry. In conclusion, parents’ awareness of amblyopia in Tabuk City, KSA, was low. In addition, a limited proportion of parents reported consistently taking their children for routine eye exams. Therefore, raising awareness should be considered in public education regarding the disease.

## 1. Introduction

Amblyopia is a reduced best-corrected visual acuity of one or both eyes that cannot be attributed to a structural abnormality [1,2]. It is considered the leading cause of visual defects in children [3,4]. Amblyopia reflects a disturbance of the brain’s visual development, which results in neural impairments caused by uncorrected refractive errors, strabismus, or rarely deprivation [5]. Amblyopia is common in the range between infancy and eight years of age [6,7,8]. It is classified as monocular or binocular, without physical or pathologic abnormalities [9]. Monocular amblyopia is mainly caused by anisometropia and strabismus [10]. High uncorrected refractive errors cause binocular amblyopia. Amblyopia is associated with abnormal eye movements, poor accommodation, abnormal contour interactions, fixation instability, reduced contrast sensitivity, and binocular dysfunction [11,12,13,14,15,16,17,18,19,20,21]. Amblyopia can also lead to psychological complications such as depression and low self-esteem. In addition, it can cause poor school performance and predict future difficulties in attaining jobs [22,23]. Very good school performance in children with severe amblyopia can be achieved when the family and society are involved in their treatment journey [24]. Amblyopia treatment consists of patching the good eye to enforce using the affected eye, and some studies showed a significant improvement with atropine [25,26,27]. The success rate of patching ranges from 49% to 87%. However, it is mainly dependent on patient compliance [28,29]. Other treatment modalities include performing visual exercises that promote recovery in the visual acuity [30].

The estimated prevalence of amblyopia worldwide is approximately 1.75% [31]. Different reports have estimated the prevalence of amblyopia in areas of Saudi Arabia. The prevalence of amblyopia among preschool children in Riyadh and Jeddah was 0.5% and 1.3%, respectively. In primary school children, amblyopia prevalence was recorded in Al Hassa (1.4%), Abha (1.85%), and Al Qassim (3.9%) [6,32,33,34,35].

Despite the available diagnostic methods for amblyopia, it is still underreported [36]. Therefore, it is critical to diagnose and treat amblyopia early to achieve the best outcomes, which can be accomplished by routine eye exams. In Saudi Arabia, children are only mandated to have an eye screening test as a requirement for elementary school entry (after the age of five years). Health practitioners also perform eye health screening for children at birth, three and five years of age. Other practitioners prefer to adopt the vision screening recommendations of the American Academy of Pediatrics (AAP) and American Academy of Pediatric Ophthalmology and Strabismus (AAPOS) that recommend an age-appropriate screening with referral criteria [2,37,38,39,40,41].

Since amblyopia is mainly a childhood disease, parents need adequate knowledge and awareness of the disorder to seek medical attention and achieve the best outcomes. Parents are usually the first to notice any changes or abnormalities in their children’s eyes appearance, alignment, and movement, which prompt them to seek medical advice to address these concerns. Additionally, parents of amblyopic children play a significant role in ensuring the compliance of their children’s treatment plan and follow-up appointments adherence with the ophthalmology clinic. Thus, measuring the parents’ awareness level is crucial, as the lack of knowledge among parents regarding eye health leads to delays in obtaining the recommended eye care at the appropriate time [37,42,43,44].

Previous studies have measured the level of parents’ knowledge and awareness of amblyopia. The parental awareness level of ocular diseases such as amblyopia and strabismus in Europe and North America was reported to be moderate [43,44,45]. In other countries, parents were not as aware of amblyopia as other ocular diseases like cataract and strabismus [46,47]. In Saudi Arabia, only a few studies were conducted to measure the level of awareness of amblyopia in parents. One conducted in Jeddah reported that most parents showed poor knowledge of amblyopia regarding many aspects, such as the correct definition, treatment options, and possible causes [48]. A report from Al Hassa showed a mild to moderate level of knowledge of amblyopia, and most of the participants acknowledged the role of parents in detection and prevention of amblyopia [49]. Another study was conducted in different regions of Saudi Arabia and reported that parents have insufficient amblyopia awareness. However, this study represented the whole northern region in one group with only 85 participants [38].

To our knowledge, this report is the first to investigate the level of awareness of amblyopia among parents in Tabuk city, Saudi Arabia. Our main objective is to determine the awareness level of amblyopia among parents in Tabuk. Also, we hypothesize that mothers are more aware of the disease than fathers, parents are not compliant with regular eye screening visits, and parents of children with eye diseases have a better knowledge of amblyopia than those with healthy children.

## 2. Materials and Methods

### 2.1. Study Design

This study is a quantitative, non-experimental, cross-sectional, prospective, descriptive study using self-administered electronic questionnaires.

### 2.2. Sample

We used an online sample size calculator, Raosoft, Inc. (2004), Seattle, United States [50]. A confidence interval of 95%, a 5% margin of error on a population of 600,000, and a response distribution of 50% were chosen. The representative sample size was 384 participants.

### 2.3. Instruments

The questionnaire was obtained, with a few modifications, from previously published research with similar aims [51] and was divided into five sections. The first section (11 questions) included demographics (age, gender, marital status, occupation, residential area, educational level, history of eye diseases in any of the children, family history of eye diseases, and history of amblyopia). The second section (three questions) evaluated the level of amblyopia awareness regarding its definition and causes; each of these questions had multiple choices, and participants could choose more than one option. The third section (eight questions) assessed the level of awareness regarding amblyopia symptoms, signs, diagnostic methods, and trusted sources of information, where participants could choose yes, no, or “I do not know” options to respond to signs, symptoms, and diagnosis questions, while in the “trusted source of information” question, the participants could choose more than one option. The fourth section (four questions) measured the knowledge of possible complications and treatment options, where yes, no, or I do not know options were given to the participants to choose from. Finally, the fifth section (eight questions) evaluated the level of awareness regarding the role of parents, where eight elements that parents may play a role in were offered, and subjects could choose between strongly agree, agree, kind of agree, disagree, and strongly disagree. In addition, two screening questions were used, “Are you living in Tabuk city?” and “Do you have children?” Those who lived in Tabuk city and had children were allowed to complete the survey.

A pilot sample of ten participants was randomly selected to test the leading and complex questions; subsequently, these ten subjects were excluded from the final analysis.

### 2.4. Procedure

Approval for the study was obtained through the research ethics committee in the University of Tabuk, approval number (READ0085) on 19 May 2020. Informed consent was obtained electronically from the participants after explaining the aims of the study.

The questionnaire was distributed by regional news and advertisement organizations through their highly followed social media accounts (Twitter and Snapchat). Participation in the questionnaire was advertised and encouraged by famous local journalists and influencers using their social media accounts to ensure they reached parents living in Tabuk city, Saudi Arabia.

### 2.5. Data Analysis

The primary outcome was participants’ responses regarding awareness of amblyopia. To score the participants’ responses, we used binary coding based on the following scoring criteria: correct answer = 1 and wrong answer = 0, in each of the 15 questions (from the second, third, and fourth sections of the questionnaire) that assess the knowledge level. For Yes/No questions, a correct answer was given a score of 1, and an incorrect answer received a score of 0. (An incorrect answer included both the wrong and the “I do not know” options). For multiple-choice questions, where choices could include more than one correct option and subjects could choose more than one answer, if a participant chose half or more of the correct options (50% or higher), the score would be 1, even if other incorrect options were also selected. Conversely, if the participant chose fewer than half of the correct options, the score would be 0. For example, in the question on the definition of amblyopia, which includes seven options, three of them are correct; if a participant chose two or more of the correct options, the score for this question would be 1. Since each question has a possible score of 1, the final score for each participant could range from 0 to 15. Then, based on their total score out of 15, we classified the subjects’ awareness level into three categories (good, fair, and poor). Thus, (0–7) correct answers was classified as a “poor” level of awareness, (8–11) correct answers was considered a “fair” level of awareness, and (12–15) correct answers was classified as a “good” level of awareness. For the analysis of the question regarding the role of parents (fifth section), we used the Likert scale [52] in which participants specify their level of agreement or disagreement (strongly agree, agree, kind of agree, disagree, strongly disagree) on a symmetric agree–disagree scale for a series of eight statements.

For data analysis, a univariate analysis was performed using the Chi-square test of association (X²) to assess whether the awareness score (three levels) was significantly different from the expected hypotheses. In addition, two-tailed hypothesis testing was performed, and a significance level of 0.05 was used throughout the analysis [53]. All the analyses were performed using the Statistical Package of SPSS v.25.

## 3. Results

The responses of 325 out of targeted 384 parents from Tabuk were received, consisting of 209 mothers (64.3%) and 116 fathers (35.7%); 200 participants (61.5%) were in the age group of 35–50 years, 76 (23.4%) were in the 20–34 age group, and 49 parents (15.1%) were older than 50 years of age; 294 participants were married (90.5%), 25 separated (7.7%), and six widows/widowers (1.8%). Regarding occupational status, 202 parents were employed (62.2%), 66 retired (20.3%), 28 housewives (8.6%), 12 unemployed (3.7%), and 17 had another occupational status (5.2%). For education level, holders of a college degree numbered 219 (67.2%), master’s degree 35 (10.8), less than high school diploma 33 (10.2%), other diplomas 20 (6.2%), high school diploma 11 (3.4%), and Ph.D. holders totaled seven (2.2%). The majority of the participants or their partners, 228 (70.2%), had no history of eye diseases, while 97 (29.8%) did. Parents who had a history of amblyopia were 23 (7.1%), and 302 (92.9%) had no history of amblyopia. Our results also showed that 220 (67.7%) participants reported that none of their children had any eye diseases. In comparison, 105 (32.3%) had at least one child with an eye disease. When participants were asked if they have an amblyopic child, 39 (12%) answered “yes”, while 286 (88%) answered “no”. (Table 1)

More than half of the participants, 176 (54.2%), had never heard of amblyopia before, while 149 (45.8%) had heard of it.

Regarding the parents’ knowledge of amblyopia, the highest percentage of the correct answers was 92% for the question “Is it important to examine the visual acuity of a child before school entrance to ensure the normal development of vision?”, and approximately 17% of the participants were knowledgeable regarding amblyopia’s definition and etiologies. (Table 2).

**Figure 1:**
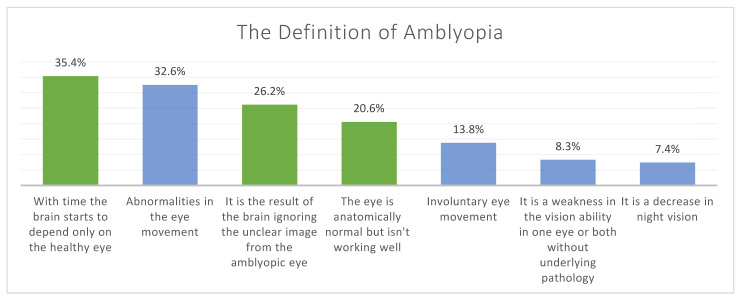
The proportions of participants’ responses for their knowledge regarding the definition of amblyopia, where they had the option to choose more than one answer. (The correct answers are represented by green bars and the incorrect answers by blue bars).

**Figure 2:**
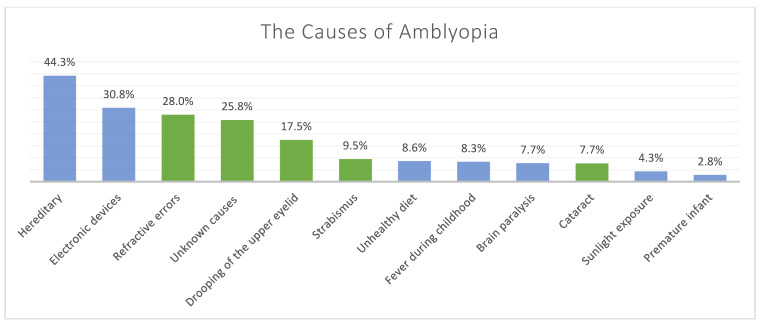
The proportions of participants’ responses for their knowledge regarding the causes of amblyopia, where they had the option to choose more than one answer. (The correct answers are represented by green bars and the incorrect answers by blue bars).

The proportions and frequencies of each chosen option for definitions and causes of amblyopia are represented in Figure 1 and Figure 2.

Among the participants, only 45 (13.8%) parents took their children for a yearly routine eye screening, 46 (14.2%) took their children only before they start elementary school, and the majority, 234 (72%), took their children for an eye screening only if they had an ophthalmic complaint.

**Figure 3:**
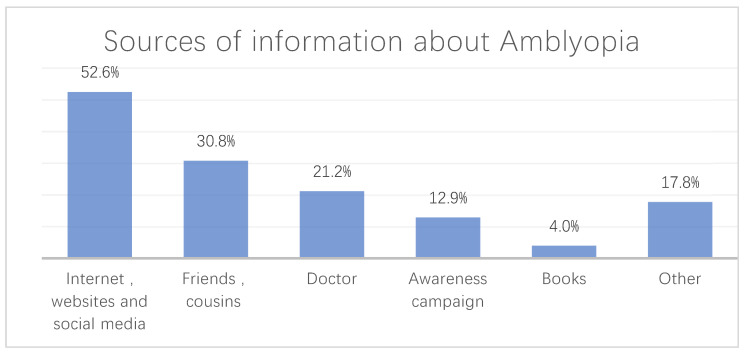
The most common sources of information regarding amblyopia.

The questionnaire included a question concerning the source of information where participants obtained their knowledge of amblyopia. The participants had the option to choose more than one source of information. The most frequent source of information regarding amblyopia chosen was “Internet, websites, and social media” (Figure 3).

When the participants were asked about the treatment of amblyopia, the most frequent chosen option was “eye exercises”; when asked about amblyopia complications, “decreases visual acuity” received the highest percentage; the most frequently chosen option for the role that parents play when having a child with amblyopia was “compliance of treatment” and the least chosen option was “preventing amblyopia from happening in the first place”.

Among the parents who had children with amblyopia (39 participants, 12%), 28 (72%) had only one amblyopic child; 17 (44%) had their children diagnosed with amblyopia after noticing an abnormality or a weird behavior such as head tilting or eye rubbing; 17 (44%) of the participants had their children diagnosed with amblyopia at the age of 1–5 years; 31 (80%) were compliant with the treatment plan; 32 (82%) reported that the disease was well-explained to them by the physician. When these parents of amblyopic children were asked if they consistently attend the follow-up visits for their children’s amblyopia treatment, approximately two-thirds (25, 64%) indicated that they do not because there is no need/benefit from these visits, nine (23%) only went to the first visit and claimed they are compliant with the treatment plan at home, and only five (13%) of the parents had attended all the follow-up appointments.

Regarding level of awareness, our results showed that 202 (62%) had a fair level of awareness of amblyopia, followed by 113 (35%) participants with a poor awareness level. A good level of awareness of amblyopia was found in only 10 (3%) participants. (Table 3).

Among the participants, a “good” level of awareness was more prominent in mothers than fathers by 4.3% and 0.9%, respectively. In addition, the “poor” level of awareness was lower among mothers (33.5%) when compared to fathers (37.1%); however, the relationship between awareness level of amblyopia and gender was not statistically significant (*p* = 0.207). (Table 4).

Results also showed that 60% of parents with a “good” level of awareness of amblyopia had children with eye diseases, and 72% of the parents with a “poor” level of awareness did not have any children with eye diseases. However, this association was also not statistically significant (*p* = 0.111). (Table 5).

## 4. Discussion

Early detection of amblyopia is crucial to avoid life-long complications. Parents’ knowledge and awareness of the disease are essential in amblyopia management [22,23,24,25,26,27,28,29,30,31,32,33,34,35,36,37,38,39,40,41,42,43,44,45,46,47,48,49]. Our study investigated amblyopia awareness and knowledge levels among parents in Tabuk City, Kingdom of Saudi Arabia. Tabuk is the biggest urban area in the north of Saudi Arabia. It is served by many hospitals, including King Khaled General Hospital, King Fahad Specialist Hospital, and Children’s Hospital; all are government-owned hospitals that provide secondary eye care and were established in 1983, 2013 and 2019 respectively. They provide mostly free services and see approximately 30,000 patients per year. In Tabuk, few studies have estimated the knowledge and awareness of eye diseases in general, and there are few studies regarding specific eye diseases [54]. Our study found that most parents had a fair awareness level (62%), while a good awareness level was found in only 3% of the participants. These results are consistent with reports in Nigeria and India (2.9% and 3%, respectively) [55,56]. However, it differs from the results of other studies conducted in Saudi Arabia. Previous assessments performed in Jeddah and Riyadh reported a good knowledge level in approximately 25.9% and 30% of participants, respectively [2,51].

Similarly, a different study conducted to measure the level of awareness among parents in five different regions of Saudi Arabia (northern, southern, western, eastern, and central) reported that 30% of participants had an adequate level of awareness regarding amblyopia [38]. Another study performed in Jeddah showed that approximately 50% of the participants were knowledgeable concerning the disease [48]. These studies highlighted a significantly higher awareness level in other parts of the country compared to Tabuk city. This observation represents the variation of knowledge levels among parents in different regions of the country. Additionally, it could be because some of their data were collected from clinic attendees and awareness campaign visitors; therefore, those parents tended to be more aware of the disorder. However, our data were collected from the general population of Tabuk using social media as a distribution method, which may be more representative of the awareness level among the general population.

The low percentage (14%) of parents who yearly took their children for routine eye exams, and the fact that most of the parents of amblyopic children believed that there was no need to attend the scheduled follow up appointments, clearly justifies the importance of establishing national vision screening guidelines and the necessity of increasing the population’s awareness level of amblyopia and other eye diseases. Furthermore, these screening guidelines could be implemented in the school system, which would take the burden off the parents.

As hypothesized, mothers were more aware of amblyopia than fathers, and parents of children with an eye disease had a higher knowledge of amblyopia than other parents. However, these two associations were not statistically significant. Furthermore, the current study showed that the most common source of information in Tabuk was the Internet and social media, followed by cousins and friends, and then physicians, which differs from the other cities’ reports. For instance, the study that included various regions of the country featured eye-care clinicians as the major source of information [38]. Moreover, a report in Jeddah indicated that physicians were also the most frequent source of information regarding amblyopia [2].

When looking at the discrepancy between the proportion of amblyopic parents (7%) and parents of amblyopic children (12%), this increase in the proportion of diagnosing amblyopia may be due to the improvement in the healthcare system and the implementation of amblyopia screening methods over the years in Tabuk.

Our study could be limited by the self-reported nature of obtaining the information, which may have led to misclassification bias if the participants did not correctly interpret some questions. Furthermore, an unavoidable bias was introduced in questions that had more than one correct answer where the participants could choose more than one option; if all the options were selected, the participant’s result would be falsely counted as a correct response, which may also have led to misclassification bias. Additionally, the subjects’ recruitment method, the limited number of participants compared to the total population of Tabuk, and the fact that most of them are females could all result in a selection bias. Finally, the representation of our results could be biased by the fact that the received responses from the participants (325) were fewer than the targeted sample size (384). Thus, population-based studies in Tabuk assessing the awareness level of amblyopia among parents are still needed to represent the population better.

## 5. Conclusions

In conclusion, our study showed a lack of awareness and knowledge of amblyopia among parents in Tabuk. We recommend implementing vision screening guidelines in the region, increasing public awareness towards amblyopia, and shedding light on the importance of regular eye exams. An adequate level of knowledge can be achieved by organizing school screening programs and educational workshops for parents given by ophthalmologists, pediatricians, or family physicians. Governmental health entities, e.g., the Ministry of Health, may also be able to play a role in raising the community’s awareness level by organizing public campaigns and awareness days in public places (e.g., shopping malls and public parks), where parents can have direct access to trusted sources of information regarding amblyopia in places outside hospitals and clinics. In addition, those health entities can also strengthen the presence of their official accounts on popular social media platforms by posting educational statements and short clips showing the importance of early detection and treatment of amblyopia.

## Figures and Tables

**Table 1:** Demographics of the sample.

		Frequency	Percent
Gender	Male	116	35.7
	Female	209	64.3
Age	20 to less than 35 years	76	23.4
	35–50 years	200	61.5
	Above 50 years	49	15.1
Marital Status	Married	294	90.5
	Separated	25	7.7
	Widow/widower	6	1.8
Occupational status	Employed	202	62.2
	Unemployed	12	3.7
	Housewife	28	8.6
	Retired	66	20.3
	Other	17	5.2
Education	Less than High school diploma/least	33	10.2
	High school diploma/least	11	3.4
	College degree	217	66.8
	Diploma	20	6.2
	Master’s Degree	35	10.8
	PhD	7	2.2
Do any of your children have amblyopia?	No	286	88
	Yes	39	12
Do you or your partner have any eye diseases?	No	228	70.2
	Yes	97	29.8
Do any of your children have eye diseases?	No	220	67.7
	Yes	105	32.3
Have you been diagnosed with amblyopia before?	No	302	92.9
	Yes	23	7.1

**Table 2:** Participants’ responses towards awareness of amblyopia.

		Correct Answers	%
		*n*	
Awareness	What is the definition of amblyopia?	56	17.3%
	What are the causes of amblyopia?	54	16.7%
	Which age group can amblyopia affect?	226	69.8%
	The eye of my child externally looks healthy, so is there a need for an eye examination?	144	44.4%
	Most cases are discovered accidentally, so is it essential for an ophthalmologist to screen and examine the child’s eye?	280	86.4%
	Can a pediatrician diagnose amblyopia?	116	35.8%
	Does closing the eyes for a short time or pressing them while watching TV considered a sign that indicates the possibility of amblyopia?	166	51.2%
	Is it difficult for parents to notice this problem because the child cannot know that his/her vision is weak?	174	53.7%
	Is it essential to examine the child’s visual acuity before school entrance to ensure the normal development of vision?	299	92.3%
	What is the recommended number of vision screenings for a child aged (6–12) years?	181	55.9%
	Do you take your child for a routine vision screening?	45	13.9%
	Is there a treatment for amblyopia?	183	56.5%
	Do you think the treatment must be at an early age?	248	76.5%
	What is the best age range to treat amblyopia?	110	34.0%
	Do you think amblyopia becomes worse if left untreated at an early age?	256	79.0%

**Table 3:** Level of awareness of amblyopia among parents in Tabuk, Saudi Arabia.

Level of Awareness	*n*	%
Poor	113	35
Fair	202	62
Good	10	3
Total	325	100

**Table 4:** The relationship between “awareness levels of amblyopia among parents” and “gender”.

Gender	Level of Awareness				
	Poor *n* (%)	Fair *n* (%)	Good *n* (%)	Total *n* (%)	*p*-Value *
Male	43 (37.1)	72 (62)	1 (0.9)	116 (100)	0.207
Female	70 (33.5)	130 (62.2)	9 (4.3)	209 (100)	

* Significance level was set at 0.05.

**Table 5:** The relationship between “awareness levels of amblyopia among parents” and “having a child with an eye disease”.

	Level of Awareness			
	Poor *n* (%)	Fair *n* (%)	Good *n* (%)	*p*-Value *
The Presence of Eye Diseases in Children				
Yes	32 (28.3)	67 (33.2)	6 (60)	0.111
No	81 (71.7)	135 (66.8)	4 (40)	
Total *n* (%)	113 (100)	202 (100)	10 (100)	

* Significance level was set at 0.05.

## Data Availability

All the data presented in this manuscript are available on request.
